# LncPCD: a manually curated database of experimentally supported associations between lncRNA-mediated programmed cell death and diseases

**DOI:** 10.1093/database/baad087

**Published:** 2023-11-27

**Authors:** Ni He, Danyang Li, Fanfan Xu, Jingnan Jin, Lifang Li, Liting Tian, Biying Chen, Xiaoju Li, Shangwei Ning, Lihua Wang, Jianjian Wang

**Affiliations:** Department of Neurology, The Second Affiliated Hospital of Harbin Medical University, Baojian Road, Nangang District, Harbin, Heilongjiang 150081, China; Department of Neurology, The Second Affiliated Hospital of Harbin Medical University, Baojian Road, Nangang District, Harbin, Heilongjiang 150081, China; Department of Neurology, The Second Affiliated Hospital of Harbin Medical University, Baojian Road, Nangang District, Harbin, Heilongjiang 150081, China; Department of Neurology, The Second Affiliated Hospital of Harbin Medical University, Baojian Road, Nangang District, Harbin, Heilongjiang 150081, China; Department of Neurology, The Second Affiliated Hospital of Harbin Medical University, Baojian Road, Nangang District, Harbin, Heilongjiang 150081, China; Department of Neurology, The Second Affiliated Hospital of Harbin Medical University, Baojian Road, Nangang District, Harbin, Heilongjiang 150081, China; Department of Neurology, The Second Affiliated Hospital of Harbin Medical University, Baojian Road, Nangang District, Harbin, Heilongjiang 150081, China; College of Bioinformatics Science and Technology, Harbin Medical University, Baojian Road, Nangang District, Harbin, Heilongjiang 150081, China; Department of Neurology, The Second Affiliated Hospital of Harbin Medical University, Baojian Road, Nangang District, Harbin, Heilongjiang 150081, China; Department of Neurology, The Second Affiliated Hospital of Harbin Medical University, Baojian Road, Nangang District, Harbin, Heilongjiang 150081, China; Department of Neurology, The Second Affiliated Hospital of Harbin Medical University, Baojian Road, Nangang District, Harbin, Heilongjiang 150081, China

## Abstract

Programmed cell death (PCD) refers to controlled cell death that is conducted to keep the internal environment stable. Long noncoding RNAs (lncRNAs) participate in the progression of PCD in a variety of diseases. However, no specialized online repository is available to collect and store the associations between lncRNA-mediated PCD and diseases. Here, we developed LncPCD, a comprehensive database that provides information on experimentally supported associations of lncRNA-mediated PCD with diseases. The current version of LncPCD documents 6666 associations between five common types of PCD (apoptosis, autophagy, ferroptosis, necroptosis and pyroptosis) and 1222 lncRNAs in 331 diseases. We also manually curated a wealth of information: (1) 7 important lncRNA regulatory mechanisms, (2) 310 PCD-associated cell types in three species, (3) detailed information on lncRNA subcellular locations and (4) clinical applications for lncRNA-mediated PCD in diseases. Additionally, 10 single-cell sequencing datasets were integrated into LncPCD to characterize the dynamics of lncRNAs in diseases. Overall, LncPCD is an extremely useful resource for understanding the functions and mechanisms of lncRNA-mediated PCD in diseases.

**Database URL:**  http://spare4.hospital.studio:9000/lncPCD/Home.jsp

## Introduction

Cell death is a fundamental physiological process and is crucial for the regulation of embryonic development, organ maintenance, ageing, immunity and pathological conditions (1). Cell death includes accidental cell death (ACD) and regulatory cell death (RCD) processes. ACD is an uncontrollable biological process, whereas RCD is a controlled process involving specific signal transduction pathways and relying on dedicated molecular mechanisms. When RCD occurs under physiological conditions, it is referred to as programmed cell death (PCD) (2–4). PCD is a complex, precise, genetically controlled cellular process that plays important roles in host defence against pathogens and in maintaining homeostasis (5). During the past few decades, many forms of PCD have been identified, including apoptosis, necroptosis, autophagy, ferroptosis and pyroptosis (6, 7). Recent studies have demonstrated that excessive activation or resistance of the PCD pathway is associated with the development of disease. For example, monotropein alleviates the progression of osteoarthritis by inactivating the NF-κB pathway, leading to apoptosis and pyroptosis of chondrocytes (8). Dysregulation of PCD can lead to the development of diseases; therefore, it is necessary to investigate the potential factors that influence PCD. The process of PCD is influenced by several factors, and recent studies have found that some small molecules, such as lncRNAs, can also regulate PCD (9).

LncRNAs are transcripts longer than 200 nucleotides that are not translated into proteins. Although they are not translated into proteins, lncRNAs are nevertheless regarded as functional molecules since they regulate the expression of genes at the chromatin, transcriptional and posttranscriptional levels (10, 11). According to current studies on lncRNAs, these molecules play a significant role in the regulation of gene expression and participate in the regulation of PCD. For instance, vincristine-induced ferroptosis and apoptosis are promoted by LINC00618, and LINC00618 accelerates ferroptosis in a manner dependent upon apoptosis (12). In addition, the relationship between lncRNAs and PCD is of great significance for diseases, especially cancer, joint disease and cerebrovascular disease. For example, overexpression of GAS5 significantly inhibits triple-negative breast cancer (TNBC) cell proliferation and promotes apoptosis, thereby partially disrupting the tumour-promoting effect of ectopic expression of miR-196a-5p to inhibit TNBC progression (13). In addition, mesenchymal stem-cell-derived exosome (MSC-Exo)-mediated KLF3-AS1 represses autophagy and apoptosis in chondrocytes by activating the PI3K/Akt/mTOR signalling pathway to improve the progression of osteoarthritis (14). Furthermore, the MEG3/miR-485/AIM2 axis activates caspase1 signalling during cerebral ischaemia/reperfusion, contributing to pyroptosis, indicating that this axis may be a promising therapeutic target in ischaemic stroke (15). There is increasing evidence that lncRNA-mediated PCD is closely related to disease. Therefore, it is necessary to systematically collect and collate information on the associations between diseases and lncRNA-mediated PCD.

To date, several PCD-related databases have been developed. For example, the database DeathBase contains information on proteins related to apoptosis and other types of cell death (16). The Autophagy Database offers structural and functional details on a wide range of autophagy-related proteins that can be used to study various species (17). HAMdb is a database of human autophagy modulators with specific pathway and disease information (18). FerrDb and FerrDb V2 are databases of regulators and markers of ferroptosis and ferroptosis-disease associations (19, 20). Unfortunately, there is currently no resource devoted to compiling the most recent and empirically verified associations between lncRNA-mediated PCD and diseases.

To address this knowledge gap, we developed LncPCD, a brand new database that gathers and combines information on the connections between both lncRNA-mediated PCD and diseases into a comprehensive resource. The LncPCD currently documents the following (1): 6666 associations between lncRNAs and five common types of PCD in 331 diseases (2), 7 lncRNA regulatory mechanisms involving 1222 LncRNAs (3), 310 PCD-associated cell types in three species (4), important cell death signal pathways and clinical applications (diagnosis, therapy, prognosis) for lncRNA-mediated PCD in diseases (5), detailed information on lncRNA subcellular locations and (6) 10 single-cell sequencing datasets integrated into LncPCD to characterize the dynamics of lncRNA in diseases. We expect that this comprehensive database, created specifically to examine the relationship between lncRNA-mediated PCD and diseases, will serve as an important impetus for further investigation.

## Materials and methods

### Data collection

**Figure 1.**
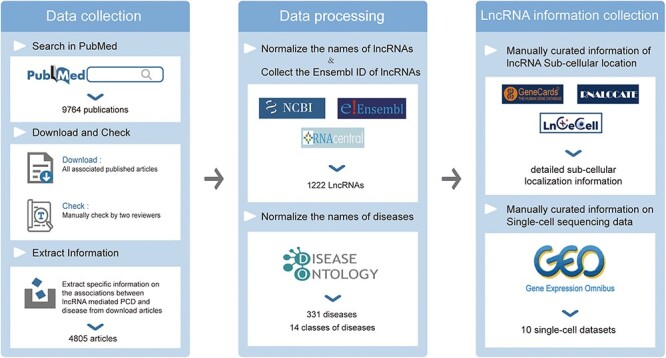
Data collection and processing of the LncPCD database.

To collect experimentally supported associations between lncRNA-mediated PCD and diseases and to ensure the quality of the database, all LncPCD entries were manually collected through several steps, as previously mentioned (21, 22). The data collection and processing of the LncPCD database are shown in Figure 1. First, we searched the literature in the PubMed database (https://pubmed.ncbi.nlm.nih.gov/) using the keywords ‘long non-coding RNA’,‘lncRNA’ or ‘lincRNA’ in combination with five common and widely studied types of PCD: ‘apoptosis’, ‘autophagy’, ‘ferroptosis’, ‘necroptosis’ or ‘pyroptosis’. PubMed articles were identified by searching these keywords in the full-text articles on PubMed. Over 9764 published studies were identified from the PubMed database. Next, we downloaded all published articles expounding the associations between lncRNA-mediated PCD and diseases, and all selected studies were reviewed by at least two researchers. Second, we manually retrieved entries related to lncRNA-mediated PCD and disease associations. In this step, we gathered information on the lncRNA name, disease name, cell death processes, species, lncRNA regulatory mechanism, lncRNA target, cell type, cell death pathway, clinical application, experimental techniques and lncRNA expression patterns. We also gathered information about the original article (PubMed ID, year of publication, title of paper) and a brief description of the role of lncRNA-mediated PCD in the original article. We collected only high-quality associations with strong experimental evidence that were experimentally confirmed by Western blot, qRT‒PCR or luciferase reporter assays. Through strict screening, 4805 articles were determined to contain relevant information that was incorporated into the database. Preliminary data regarding the connection between lncRNA involvement in PCD and disease were gathered through the aforementioned steps.

### Data processing

The preliminary information gathered from the literature was arranged and processed carefully. First, we verified the lncRNA names in all selected studies, and the names of the lncRNAs were altered to the official or recommended names. When the lncRNAs were not exactly the same as the Ensembl entries, we used the lncRNA names mentioned in the original articles. In this step, we also collected the Ensembl ID information of lncRNAs for LncPCD by searching the NCBI Gene (https://www.ncbi.nlm.nih.gov), RNAcentral 2021 (23) and Ensembl 2022 (24) databases. Second, we used a standardized classification scheme, the Disease Ontology (25), to standardize disease names and classifications. Third, to better describe the relationship between lncRNAs and PCD, we collected subcellular localization information of lncRNAs from the GeneCards (https://www.genecards.org/), LnCeCell (26) and RNALocate v2.0 (27) databases. In addition, we thoroughly collected human cancer single-cell RNA-seq (scRNA-seq) datasets from The Gene Expression Omnibus (GEO) database to characterize the expression of lncRNAs (http://www.ncbi.nlm.nih.gov/geo). Information on scRNA-seq data, including different cancer types, cell lines and cell numbers, was also extracted. A total of 10 single-cell datasets across 9 cancer types were finally obtained. The raw single-cell data were downloaded from https://www.ncbi.nlm.nih.gov/sra/. For 10X data, the processes of alignment, cell barcode demultiplexing, transcript quantification and sample merging were performed with CellRanger 7.0.0 using the hg38 human reference genome file (refdata-cellranger-GRCh38-3.0.0) with the default options and parameters. For SMART-seq data, alignment was conducted using STAR-v2.7.9a, and then the expression profiles of the cells were annotated based on GENCODE (release 34, GRCh38). Finally, the relative gene expression was quantified as transcripts per million (TPM) using RSEM v1.2.17. After the upstream processing above, the processed count data (10X data) and the input TPMs transformed by log2 (TPM + 1) were subjected to the standard Seurat procedure. To reduce the effects of high technical noise in single-cell expression profiles, we performed quality control on the downstream single cells: we excluded cells that expressed fewer than 200 genes and retained genes with detectable expression in at least 1% of cells.

### Database construction

All data in the LncPCD were documented and managed using the MySQL (V 5.5) system. The web interface was built using Java Server Pages. The scripts for the data processing programs were written in Java. The creation of results tables and visualization of data were performed using jQuery (V1.11.1), Datatable (V1.10.4) and ECharts (V4.0) plugin software. The web service runs on an Apache Tomcat web server (V6). The LncPCD is freely available at http://spare4.hospital.studio:9000/lncPCD/Home.jsp.

## Results

### Data statistics

**Figure 2.**
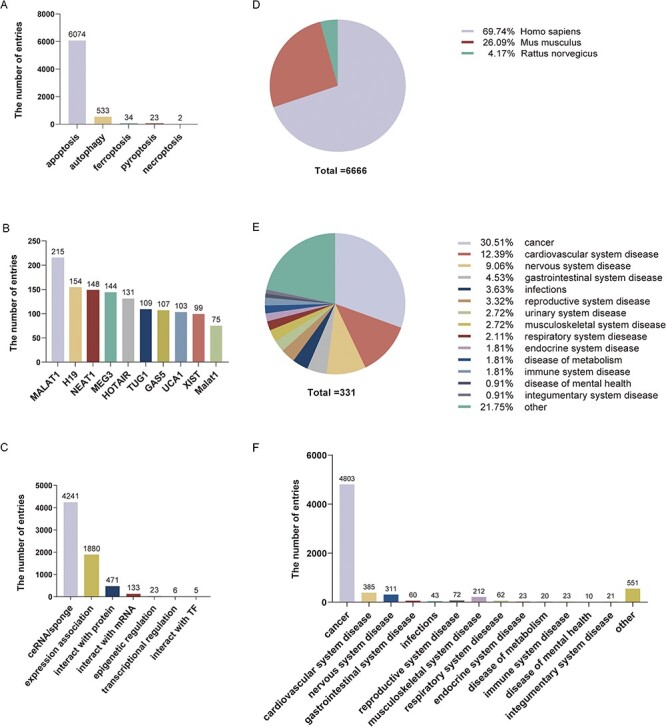
Statistical information on LncPCD. (A) Numbers of entries for different types of programmed cell death. (B) Numbers of entries for the top 10 lncRNAs. (C) Numbers of entries for lncRNA-mediated regulatory mechanisms. (D) Numbers of entries for three species. (E) Numbers of diseases for different classes. (F) Numbers of entries in different disease classes.

The current version of LncPCD contains 6666 experimentally supported associations between 1222 lncRNAs and five common types of PCD in 310 diseases (Table 1). In total, we curated five common types of PCD, including apoptosis (6074 entries), autophagy (533 entries), ferroptosis (34 entries), pyroptosis (23 entries) and necroptosis (2 entries) (Figure 2A). This suggests that investigation of lncRNA-mediated PCD in the development of diseases is one of the most popular topics in biomedical research. However, the above five types of specific entries showed different patterns. The number of apoptosis entries increased slowly from 2011 to 2016 but then increased dramatically from 2017 to 2022. In addition, the numbers of autophagy, ferroptosis, pyroptosis and necroptosis entries have increased in the recent years. In contrast, the numbers of entries related to relationships between lncRNA-mediated ferroptosis, pyroptosis, necroptosis and disease increased after 2016, suggesting that these types of PCD will be a focus of research in the future (Supplementary material Table S1).

**Table 1.** The statistics information of LncPCD

Element	Number
Total entry	6666
LncRNA	1222
Disease	331
LncRNA RM	7
PCD type	5
Cell type	310
Species	3
Single-cell sequencing dataset	10

As mentioned above, LncPCD contains 1222 lncRNAs, and the top 10 key lncRNAs ranked by the number of entries included MALAT1 (215 entries), H19 (154 entries), NEAT1 (148 entries), MEG3 (144 entries), HOTAIR (131 entries), TUG1 (109 entries), GAS5 (107 entries), UCA1 (103 entries), XIST (99 entries) and Malat1 (75 entries) (Figure 2B). Furthermore, LncPCD records entries related to 7 types of significant lncRNA regulatory mechanisms, including ceRNA or sponge (4241 entries), expression association (1880 entries), interaction with protein (471 entries), interaction with mRNA (133 entries), epigenetic regulation (23 entries), transcriptional control (6 entries) and interact with TF (5 entries) (Figure 2C). Additionally, LncPCD lists 310 cell types associated with PCD in *Homo sapiens, Mus musculus* and *Rattus norvegicus*; the numbers of species-related entries are shown in Figure 2D.

In total, we curated 331 diseases in LncPCD, of which, cancer (30.51%), cardiovascular system disease (12.39%) and nervous system disease (9.06%) were the top three classes (Figure 2E). In addition, we observed 4803 cancer-related entries, accounting for 72.05% of the total number of entries in LncPCD (Figure 2F). These observations show that lncRNA-associated PCD has been studied in a variety of diseases and is especially widely studied in cancer. Previous studies have discovered several types of PCD that play a crucial role in regulating the immunosuppressive tumour microenvironment (TME) and in determining the clinical outcome of cancer therapies (28). Thus, PCD plays an important role in cancer progression and immune regulation and is a promising therapeutic strategy for cancer.

### User interface

**Figure 3.**
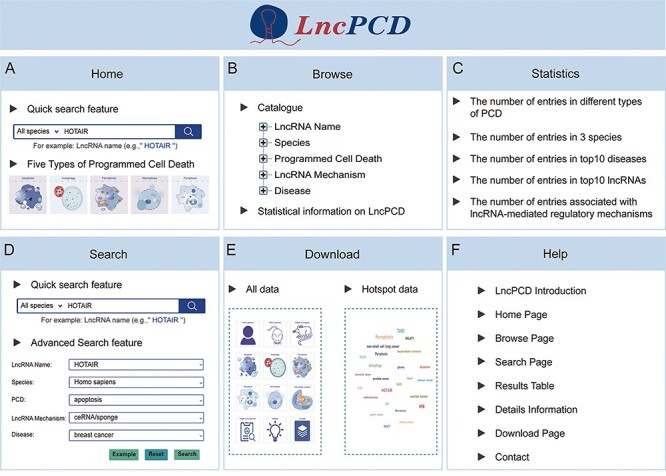
Overview of the LncPCD web interface. (A) Overview of the Home page. (B) Overview of the Browse page. (C) Overview of the Statistics page. (D) Overview of the Search page. (E) Overview of the Download page. (F) Overview of the Help page.

LncPCD is publicly available at http://spare4.hospital.studio:9000/lncPCD/Home.jsp. LncPCD provides a user-friendly web interface that allows intuitive browsing, searching and downloading of all associations between lncRNA-mediated PCD and diseases in the database. On the Home page, LncPCD offers the Quick Search feature, which allows users to select all species or particular species types (e.g. *Homo sapiens*) from the species drop-down menu. After setting the species type, users can type the lncRNA of interest (e.g. HOTAIR) to query-related associations in the LncPCD. In addition, five clickable pictures with morphological characteristics for the five types of PCD are provided. Users can click pictures of interest to view the associations between lncRNA-mediated PCD and diseases (Figure 3A). The Browse page was built based on a standardized classification scheme of diseases (according to the Disease Ontology database) and different hierarchical classifications. Users can browse the database by clicking the name on the tree view. To browse the entries related to the disease of interest, users can click ‘Disease’ and select the disease of interest. Users can browse all entries associated with an event of interest (lncRNA name, species, PCD and lncRNA mechanism) in a similar way. In addition, the Browse page provides statistical information on lncRNA-associated PCD in diseases (Figure 3B). On the Statistics page, LncPCD provides more specialized and detailed statistical information on lncRNA-associated PCD in diseases (Figure 3C). On the Search page, LncPCD provides a Quick Search panel and Advanced Search panel for searching experimentally supported associations about lncRNA-mediated PCD in diseases. The Quick Search panel is identical to the Quick Search on the home page. The Advanced Search feature provides multiple options for a customized search of the content of interest. Users can query the associations between lncRNA-mediated PCD and diseases by combining different keywords, including the lncRNA name, species, type of PCD, lncRNA regulatory mechanism and disease (Figure 3D). On the download page, LncPCD provides a downloadable file in TEXT format. The data available for download include *Homo sapiens*-associated data, *Mus musculus*-associated data, *Rattus norvegicus*-associated data, data associated with the five types of PCD, LncRNA subcellular localization information, single-cell sequencing datasets, hotspot data and all data. On the left side of the page, LncPCD provides a representative visualization map that displays all data. Users can download corresponding data by clicking on the appropriate picture. On the right side of the page, LncPCD also provides a visual map of the tag cloud that displays hotspot data. Users can download hotspot data by clicking on the appropriate area (Figure 3E). On the Help page, we provide a detailed tutorial for users on how to use LncPCD. At the bottom of the Help page, we also provide a contact function for users to contact us if they have any questions about using the website (Figure 3F).

### Data query and results display

**Figure 4.**
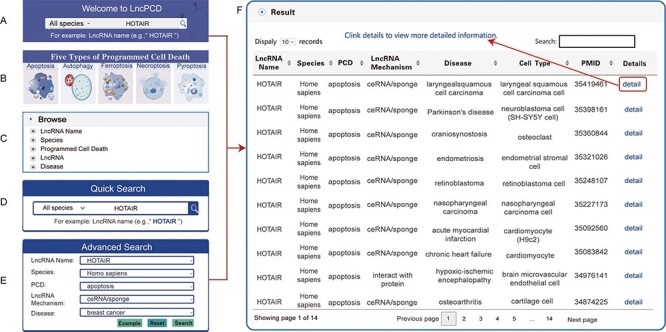
Search methods and results in the LncPCD database. There are five methods to obtain related information. (A) Quick search function on the Home page. (B) Image links to the five types of PCD on the home page. (C) Tree view function on the left side of the Browse page. (D) Quick Search function on the Search page. (E) Advanced Search function on the Search page. Through each of the above methods, a result table is obtained. (F) LncPCD results are organized in a data table, with a single association record on each line.

**Figure 5.**
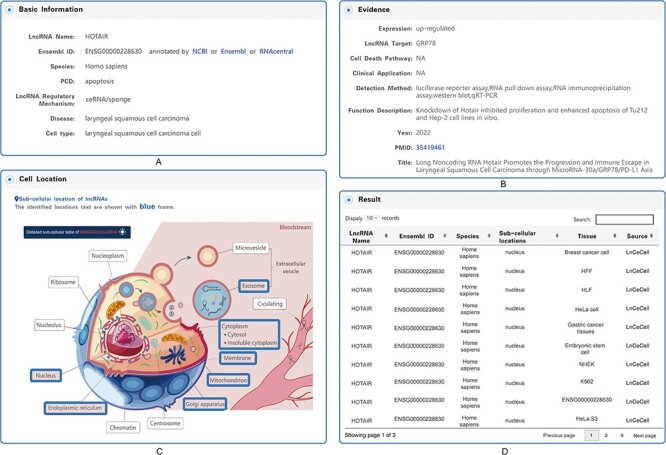
Detailed information for each entry. (A) Basic information on the associations between lncRNA-mediated PCD and diseases. (B) Evidence of the associations between lncRNA-mediated PCD and diseases. (C) Subcellular locations of lncRNAs. (D) Table of the detailed information on subcellular locations.

**Figure 6.**
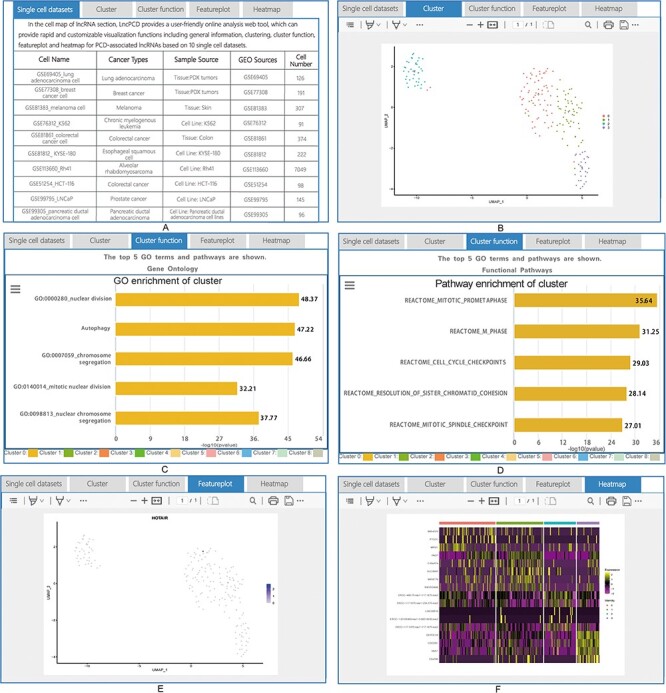
Detailed information for cell map of lncRNA. (A) General information for the single-cell sequencing data. (B) Cluster plot of lncRNAs based on single-cell sequencing data. (C) A plot of the GO enrichment of cluster. (D) A plot of the pathway enrichment of cluster. (E) Feature plot of lncRNAs based on single-cell sequencing data. (F) Heatmap plot of lncRNAs based on single-cell sequencing data.

As mentioned above, users can obtain the relevant information of interest through the Quick Search function (Figure 4A) and the image links of the five types of PCD (Figure 4B) on the Home page, the tree view on the left side of the Browse page (Figure 4C) and the Quick Search (Figure 4D) and Advanced Search (Figure 4E) functions on the Search page. The Search and Browse results in LncPCD are organized in a data table, with a single association record on each line: lncRNA name, species, PCD, lncRNA mechanism, disease, cell type, PMID, details (hyperlinks that can be clicked to get more detailed information) (Figure 4F). To further learn about and explore the associations between lncRNA-mediated PCD and diseases, users can click ‘Details’ in the result table to obtain comprehensive information. The basic information includes the lncRNA name, Ensembl ID (each lncRNA Ensembl ID hyperlinks to authoritative annotation databases), species, PCD, lncRNA regulatory mechanism, disease and cell type (Figure 5A). The evidence includes the expression, lncRNA target, cell death pathway, clinical application, detection method, function description, year, PMID and title (Figure 5B). In the cellular localization section, LncPCD provides all possible subcellular locations of the retrieved lncRNA, and the identified locations are marked with blue frames. In addition, users can obtain detailed subcellular location information by clicking on the ‘Detailed Subcellular Table’ button (Figure 5C). The detailed information includes the lncRNA name, Ensembl ID, species, subcellular location, identified tissue or cell line and data source (Figure 5D) (4). In the cell map of the lncRNA section, LncPCD provides an scRNA-seq web tool and a user-friendly online analysis module, which can provide rapid and customizable visualization functions of PCD-associated lncRNAs based on human cancer single-cell transcriptome data collected from the GEO database (Figure 6A). This online tool is equipped with four main functions, clustering, cluster function, feature plotting and heatmap plotting, for examining the expression patterns of lncRNAs. The clustering feature allows users to perform cell cluster analysis on single-cell transcriptome expression data using UMAP dimensionality reduction methods (Figure 6B). What’s more, the cluster function provides a cluster plot of the GO enrichment of the cluster (Figure 6C) and the pathway enrichment of the cluster (Figure 6D) in the ‘lncRNA relatively highly-expressed’ sub-cluster. Moreover, the Featureplotting feature enables users to obtain the expression patterns of PCD-associated lncRNAs in diverse scRNA-seq datasets (Figure 6E). Finally, the heatmap plotting feature provides a heatmap of differentially expressed transcriptomes among diverse clusters (Figure 6F). All of the above functions are performed using the R package Seurat (version 4.3.0).

## Discussion and conclusion

Many studies have shown that lncRNAs exert a wide range of biological effects and act as potent regulators of gene expression (29). Aberrant expression of lncRNAs has been detected in various diseases (30). According to recent research on PCD, in which lncRNAs play a role, lncRNAs are closely associated with the onset of disease (31–34). Since the majority of the lncRNAs linked to PCD have been discovered in independent studies carried out over many years, careful collection of the existing information on lncRNA-mediated PCD will give researchers access to a valuable resource for studying PCD-related diseases. Recently, several databases have been established to provide information related to certain types of PCD in public repositories. However, no database integrates the information on lncRNA-mediated PCD for all five types of PCD, and the numbers of experimental records in these databases are very small. In addition, there is no database on the relationship between lncRNA-mediated PCD and disease. For example, DeathBase is a database primarily containing information on apoptotic proteins (16). The Autophagy Database provides relevant functional and structural information about multispecies autophagy proteins (17). HAMdb is a database of information on human autophagy regulators with specific pathway and disease information (18). FerrDb and FerrDb V2 are databases of regulators and markers of ferroptosis and ferroptosis-disease associations (19, 20). Most of these databases collect information on one type of PCD, with some focusing on information related to PCD proteins or PCD regulators. In summary, there is currently no database that integrates information on multiple PCD pathways associated with lncRNAs, including apoptosis, autophagy, ferroptosis, necroptosis and pyroptosis. Therefore, we developed LncPCD, a comprehensive database of experimentally supported associations of lncRNA-mediated PCD with diseases.

LncPCD has several advanced features that set it apart from previous databases. First, LncPCD contains information regarding five types of PCD, including apoptosis, autophagy, ferroptosis, necroptosis and pyroptosis. In addition, LncPCD collects information on seven types of lncRNA-mediated PCD regulatory mechanisms in disease, including ceRNA or sponge, interaction with mRNA, interaction with protein, transcriptional regulation, epigenetic regulation, interact with TF and expression association. The associations between the expression levels of PCD-associated lncRNAs and associated diseases will be useful in further investigations. LncPCD also provides clinical applications (for diagnosis, therapy and prognosis) of lncRNA-mediated PCD in 331 diseases. Finally, LncPCD provides detailed subcellular localization information for 1222 lncRNAs and 10 single-cell sequencing datasets to help characterize the dynamics of lncRNAs in diseases.

In summary, LncPCD is a comprehensive database of over 6000 associations between 310 diseases and lncRNA-mediated PCD from a considerable number of published studies. We manually collected comprehensive information on the above associations among lncRNAs, PCD and disease. Moreover, we integrated cellular localization and single-cell sequencing data into LncPCD to explore the functions and mechanisms of lncRNA-mediated PCD in diseases. LncPCD offers a simple interface for searching, browsing, visualizing and downloading comprehensive information. This, in our opinion, will enable researchers to examine the functional relevance of various forms of lncRNA-mediated PCD to the pathogenesis of disease. LncPCD is a comprehensive database of information on lncRNA-mediated PCD associated with diseases and a powerful working platform to help advance our understanding of PCD.

## Future extensions

In the future, more experimentally supported lncRNA-mediated PCD and disease associations are expected to be published, and these data will be integrated into the LncPCD database. Bioinformatic approaches are becoming increasingly important for predicting disorders related to lncRNA-mediated PCD. Consequently, the long-term goal of LncPCD is the development and integration of bioinformatic techniques for the study and forecasting of lncRNA-mediated PCD and illness correlations. In our opinion, LncPCD is a useful and timely tool for advancing knowledge of the roles and molecular processes of lncRNA-mediated PCD in disease pathogenesis, which will aid in the discovery of new and accurate biomarkers and therapeutic targets for disorders.

## Supplementary Material

baad087_Supp

## Data Availability

All data and resources of LncPCD are freely available at http://spare4.hospital.studio:9000/lncPCD/Home.jsp.
