# Supplementary material for: LncPCD: a manually curated database of experimentally supported associations between lncRNA-mediated programmed cell death and diseases
Source: Database (Oxford). 2023 Nov 27;2023:baad087. doi: 10.1093/database/baad087 (PMC10681436; doi:10.1093/database/baad087)
Supplement: baad087_Supp [file baad087_supp.zip › Table S1. The number of entries in different types of programmed cell death.docx]

Table S1. Annual number of entries in different types of programmed cell death.

| Year | apoptosis | autogragy | ferroptosis | pyroptosis | necroptosis | Total |
| --- | --- | --- | --- | --- | --- | --- |
| 2011 | 1 | 0 | 0 | 0 | 0 | 1 |
| 2012 | 6 | 0 | 0 | 0 | 0 | 6 |
| 2013 | 26 | 1 | 0 | 0 | 0 | 27 |
| 2014 | 36 | 1 | 0 | 0 | 0 | 37 |
| 2015 | 116 | 8 | 0 | 0 | 0 | 124 |
| 2016 | 172 | 9 | 0 | 0 | 0 | 181 |
| 2017 | 340 | 23 | 0 | 4 | 0 | 367 |
| 2018 | 677 | 79 | 2 | 1 | 1 | 760 |
| 2019 | 1062 | 96 | 2 | 2 | 0 | 1162 |
| 2020 | 1772 | 153 | 2 | 6 | 0 | 1933 |
| 2021 | 1594 | 162 | 20 | 6 | 0 | 1782 |
| March 2022 | 272 | 1 | 8 | 4 | 1 | 286 |
| Total | 6074 | 533 | 34 | 23 | 2 | 6666 |
